# The autumn low milk yield syndrome in Brown Swiss cows in continental climates: hypotheses and facts

**DOI:** 10.1007/s11259-023-10203-0

**Published:** 2023-08-25

**Authors:** Roman Mylostyvyi, Nicola Lacetera, Massimo Amadori, Veerasamy Sejian, João Batista Freire Souza-Junior, Gundula Hoffmann

**Affiliations:** 1 Dnipro State Agrarian and Economic University, Dnipro, 49600 Ukraine; 2 Department of Agriculture and Forest Sciences, University of Tuscia, Via San Camillo De Lellis, Viterbo, 01100 Italy; 3 Italian Network of Veterinary Immunology, Brescia, Italy; 4 Rajiv Gandhi Institute of Veterinary Education and Research, Kurumbapet, Puducherry, 605009 India; 5 ThermoBio - Research Nucleus in Applied Animal Biometeorology, Federal Rural University of Semi-Arid, Mossoro, Brazil; 6 Department of Sensors and Modeling, Leibniz Institute for Agricultural Engineering and Bioeconomy, Potsdam, Germany

**Keywords:** Brown Swiss cows, Heat stress, Milk yield, THI, Mastitis

## Abstract

Extensive research has been conducted globally on the impact of heat stress (HS) on animal health and milk production in dairy cows. In this article, we examine the possible reasons for the decrease in milk production in Brown Swiss (BS) cows during the autumn season, known as the autumn low milk yield syndrome (ALMYS). This condition has been extensively studied in high-yielding Holstein Friesian (HF) cattle and has also been observed in BS cows with a daily milk yield of around 30 kg. Our hypothesis is that the drop in milk yield and the increased prevalence of mastitis in autumn, as found in our recent studies, may be a long-term consequence of summer HS. We re-evaluate our previous findings in light of the possible manifestation of an HS-related form of ALMYS in BS cows. As milk yield, mastitis spread, and reproductive function of cows are interrelated and have seasonal dependence, we examine the consistency of our hypothesis with existing data. The significant drop in milk yield in BS cows in autumn (by 2.0–3.2 kg), as well as the threshold of milk yield decrease (temperature-humidity index of 70.7), may point in favour of the manifestation of ALMYS in BS cows, similar to HF cows. Only the percentage effect of seasonal factor (59.4%; p < 0.05) on milk yield of BS cows was significant. HS-related ALMYS provides a robust conceptual framework for diverse sets of productive and animal health data in BS cows, similar to observations in high-yielding HF cattle. However, the limitations associated with the lack of additional data (e.g. immunological indicators) suggest the need for further research to confirm ALMYS in BS breed.

## Introduction

For years, dairy farmers have observed a decline in milk yield during the autumn season (Ray et al. 1992). However, this phenomenon has been defined as the autumn low milk yield syndrome (ALMYS) in Italy (Amadori and Spelta 2021). The ALMYS is characterized by a reduced milk yield in autumn compared to spring, despite similar physiological states, lactation stages, and feeding levels under thermoneutral conditions. According to the Italian Farmer Association AIA (Associazione Italiana Allevatori), the percentage of Holstein Friesian (HF) cows producing more than 40 kg/day of milk at lactation peak is significantly lower in autumn than in spring, which results in losses of up to 2.7 kg of milk / cow / day (Amadori and Spelta 2021).

Summer has been widely associated with adverse effects on animal health, reproductive performance, and milk production (Maggiolino et al. 2020; Vitali et al. 2020). Heat stress (HS) may increase the total number of bacteria and somatic cells in milk, indicating the presence of subclinical mastitis in cows and leading to clinical signs in later periods (Colakoglu et al. 2017). Maggiolino et al. (2020) reported a peak in mastitis prevalence in cows from November to January, following the first peak in clinical cases in July due to an increased heat load. Some researchers (Cook et al. 2007) have highlighted the long-term effects of summer HS, including an increased prevalence of lameness in dairy cows associated with longer periods of standing during HS to dissipate excess heat. Tao et al. (2018) report that during the dry period, heat stress negatively affects mammary gland development by reducing mammary cell proliferation before parturition, resulting in a dramatic decrease in milk production in the subsequent lactation. In addition, heat stress in animals during the transition period was significantly associated with decreased milk yield, increased mastitis and postpartum pathology, as well as decreased survival of dairy cows (Menta et al. 2022).

The temperature-humidity index (THI) is a widely recognized indicator to characterize the severity of HS in dairy cows. For example, Akyuz et al. (2010) distinguish three levels of thermal stress based on THI values: mild stress (72–79), moderate stress (79–89), and severe stress (> 89). However, determining the THI threshold for different cattle breeds and animal populations of the same breed remains difficult due to different assumptions in literature. Ravagnolo et al. (2000) demonstrated a daily milk yield decrease by around 0.2 kg per unit of THI increase above 72. However, Bouraoui et al. (2002) found that the daily milk yield per cow was reduced by 0.41 kg for every THI unit increase above 69 and in a Polish study, an increase of THI led to decreases in daily milk yield ranging from 0.18 to 0.36 kg per THI unit (Herbut and Angrecka 2012). In addition, despite a number of publications that Brown Swiss (BS) cows are more resistant to heat stress than HF cows (Mylostyvyi et al. 2021; Cuellar et al. 2023), Maggiolino et al. (2020) were unable to determine a THI threshold for reduced milk yield, although their average THI threshold for reduced protein yield of 74 was indeed higher than that of the Holstein breed.

To investigate the possible underlying causes of ALMYS in BS cows, we considered the dysregulated, inflammatory, and metabolic memory responses of the innate immune system proposed by Amadori and Spelta (2021) in the framework of “Trained Immunity”. While the increase in laminitis may be attributed to changes in the behavior of cows during summer heat periods, the decrease in milk yield and increase in clinical mastitis after HS subsides may be due to long-term immune responses triggered by HS. This hypothesis serves as the conceptual basis underlying ALMYS in high-yielding Holstein cows. Therefore, we aimed to assess the animal health and milk production data available for BS cows and determine their consistency with HS-related ALMYS and the “Trained Immunity” hypothesis. Therefore, we had to consider many factors (e.g. seasonality, THI values, calving, conception and mastitis in the herd) that could affect cow milk yield in confirming our hypothesis.

## Materials and methods

### Experimental design, housing, and feeding

To investigate our hypothesis regarding the occurrence of ALMYS in BS cows, we conducted an analysis of weather conditions, milk production, mastitis cases, conception rates, and calving distribution for two consecutive years. We adopted an integrated approach to account for the multiple factors influencing milk yield in autumn within a specific cohort of BS cattle.

The study was conducted at a commercial dairy complex that bred 1,300 dairy cows, including BS cows, near the city of Dnipro in central Ukraine. The region has a humid continental climate with hot summers (climate type Dfa according to the Köppen climate classification). The cows were housed in naturally ventilated barns (NVBs) under loose housing conditions. Their year-round feed mix was based on corn silage and was nutritionally balanced according to the recommendations of the National Research Council (NRC 2001). The cows had free access to feeding alleys and drinking troughs. More detailed information on the cows’ housing conditions is available in our previously published study (Mylostyvyi et al. 2020). The cows were milked three times a day (at 05:00, 13:00, and 21:00 h) using DeLaval milking equipment (DeLaval, Tumba, Sweden) and an automatic cluster removal system in a 20 × 2 herringbone milking parlor.

### Recording of weather conditions

Air temperature (°С) and relative humidity (%) were collected from the nearest meteorological station, “Dnipro Airport.“ These data were freely available as archival records on the official website of the Ukrainian Hydrometeorological Centre. The livestock premises were located within a straight-line distance of 21 km from the meteorological station. Weather data ordering followed the method described previously (Mylostyvyi and Chernenko 2019). The analysis included 17,544 paired records of temperature and relative humidity, with 731 daily averages calculated from these data. The data from January 2019 to December 2020 were statistically processed, with parameters taken into account every hour, and average values calculated for the day. The temperature-humidity index (THI) was calculated according to Kibler (1964):

1 $${\rm{THI}}\,{\rm{ = }}\,{\rm{1}}{\rm{.8}}\,{\rm{ \times }}\,{\rm{T}}\, - \,\left( {{\rm{1}} - {\rm{RH/100}}} \right)\,{\rm{ \times }}\,\left( {{\rm{T}} - {\rm{14}}{\rm{.3}}} \right)\,{\rm{ + }}\,{\rm{32}}$$

where T is the air temperature in °C, and RH is the relative humidity in %.

### Records on dairy productivity of animals and the incidence of mastitis

The herd consisted of 1300 ± 57 multiparous dairy cows with an average of 3.6 ± 1.4 lactations. The number of days in milk was 157 ± 94 days. The data on milk productivity of all cows, including daily milk yield per cow (kg) and the percentage of milk fat and protein, were recorded in the DairyComp 305 herd management system for two years. Daily averages were calculated based on these data. Therefore, a total of 731 records for each production trait (average daily milk yield, milk fat and protein percentage) were recorded during the study. Clinical mastitis was diagnosed using general clinical methods of examination (examination and palpation), and its subclinical form was identified using the California Mastitis Test on the farm. All the records analyzed in this study were provided by the enterprise’s veterinary service. The average monthly incidence of mastitis was calculated as the percentage ratio of animals with mastitis to the total number of dairy cows in the herd.

### Artificial insemination of cows, pregnancy check, and calving records

Data regarding the outcomes of cow insemination were gathered at the dairy complex by scrutinizing veterinary reports. Cows were artificially inseminated via the cervical method with uterus fixation via the rectum using disposable catheters from Minitüb GmbH (Tiefenbach, Germany), and the semen was obtained from Limited Liability Company Semex Alliance (Ukraine). In the absence of natural sexual cyclicity, cows were synchronized on the 85th day after giving birth using the “Ovsynch” or “Presynch” protocols depending on their clinical condition.

To calculate the rates of conception and embryo loss, linear ultrasound echography using a 7.5 MHz transrectal transducer (Kaixin KX5200; Xuzhou Kaixin Electronic Instrument Co., Ltd, Jiangsu, China) were performed on all cows at 31–37 and 56–58 days after artificial insemination. The conception rate was determined by dividing the number of pregnant cows by the number of inseminated cows.

Calvings were observed for a two-year period at the dairy farm, where they took place in the maternity ward unit. After staying with the cow for an hour and having the first forced drinking of colostrum through the esophageal tube, the newborn calf was moved to an individual cage in the calf room. This study excluded stillborn and calf mortality cases.

### Statistical analysis

The recorded data were presented as mean values (Mean) and the standard error of the mean (SE). The relationship between characteristics (average daily milk yield and average daily temperature-humidity index) was determined using Spearman’s rank-order correlation method. Significant differences between the samples (mean monthly milk yield, conception rate, number of calvings and mastitis prevalence) were determined by the Mann-Whitney U-test (significance level of α = 0.05).

Factorial ANOVA was used to determine the percent of exposure (%) of individual factors on cow milk yield. It involved grouping (coding) the data before statistical processing. When coding, the factor “Season” was assigned a value from 1 to 4 (1 is Winter; 2 is Spring; 3 is Summer; 4 is Autumn). When the factor “Year” was coded, data were coded with the numbers 1 or 2 (2019 and 2020, respectively). The factor “Temperature-humidity index, THI” was coded according to the degree of manifestation of heat stress in dairy cattle according to the previously described principle (Mylostyvyi and Chernenko 2019): 1 is the value up to 67.9 units (comfortable conditions); 2 is the interval from 68 to 71.9 units (mild stress); 3 is the interval from 72 to 79.9 units (moderate stress); 4 are values ≥ 80 units (strong stress). Factors such as “Conception rate”, “Number of calvings” and “Mastitis prevalence” were coded 1 or 2 based on values above or below the annual average for the cow herd. The percent of exposure (%) of factors on cow milk yield was determined by the method of biometric analysis (Kovalenko et al. 2010) based on the results of ANOVA in the program Statistica 12 (StatSoft, Inc., Tulsa, OK, USA). The difference with values of p < 0.05 was considered statistically significant.

To determine the threshold value of THI causing milk yield reduction in BS cows, we used ROC-analysis with MedCalc® Statistical Software version 20.106 (MedCalc Software Ltd, Ostend, Belgium). We assumed that the response of BS cows to the increase in the THI should be similar to HF cattle, for which the threshold for milk yield reduction is THI = 72 units (Herbut et al. 2018). Based on this THI value we classified the data, i.e., assigned a value of 0 or 1 to a group, which is required for ROC-analysis. The basis of ROC analysis is the construction of the ROC curve, which is most often used to predict the results of binary classification. The resulting ROC curve reflects the ratio of true positive prediction (Sensitivity) to false positive prediction (100-Specificity) for the entire range of values of the indicator under study. The quantitative interpretation of ROC is provided by the AUC (area under ROC-curve), which is the area bounded by the ROC-curve and the axis of the proportion of false positive classifications. The higher the AUC, the better the classifier performs. Model quality was assessed based on the following AUC intervals: 0.9-1 - excellent; 0.8–0.9 - good; 0.7–0.8 - fair; 0.6–0.7 - poor; 0.5–0.6 - fail (Nahm 2022). At the same time, a value of 0.5 demonstrates the unsuitability of the chosen classification method (it corresponds to a simple guess).

## Results

### Weather conditions and their relation with milk yield

**Fig. 1:**
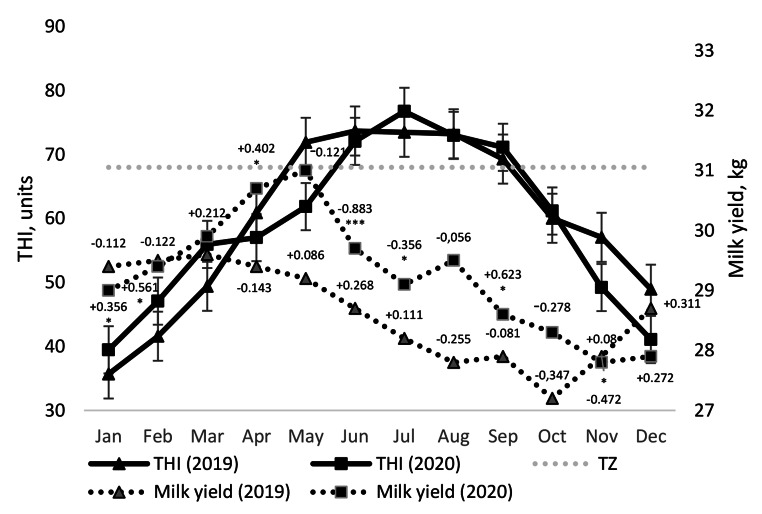
Average daily values of temperature-humidity index (THI) and mean daily milk yield in 2019 and 2020 as well as the correlation (r) between these indicators. The correlation (r) between milk yield and THI is indicated above the markers in the curves. * P < 0.05. TZ shows the upper limit of the thermoneutral zone

The temperature-humidity index was used to assess the weather conditions daily for two years. It was found that during the summer months, the average monthly THI values ranged from 66.7 to 70.3 in 2019, with the highest values observed in June. In 2020, THI values ranged from 67.1 to 69.3, with the highest value recorded in July. However, the maximum average daily THI values fluctuated from 73.2 to 73.7 in 2019 and from 72.1 to 76.8 in 2020. No significant difference was observed in the average monthly summer values of THI between the two years. It is worth noting that the maximum average daily value of THI was already 71.9 in May 2019, and in September 2020, the THI indicator (value of 71.1) exceeded the thermoneutral zone (THI < 68) for dairy cows (Fig. 1). During the autumn of 2019 and 2020, the average monthly THI values were within the comfort range for dairy cows (ranging from 42.4 to 59.6 and from 40.8 to 63.6 units, respectively). Differences between mean THI in summer and autumn were significant.

The different correlations between THI and milk yield by years (as shown in Fig. 1) may have been associated with variations in the duration of HS on the animals and the magnitude of the maximum peak values of THI during summer. This was particularly evident in the significant negative correlation between THI and milk yield in June-July 2020, which was attributed to the influence of HS. However, the apparently negative correlation between THI and milk yield in October (r=-0.28; P˃0.05) did not conform to the positive trend observed between these indicators, following a significant positive correlation in September (r = 0.62; P < 0.001) when average daily THI values returned into cow comfort levels. A similar situation occurred in the previous year (2019), with negative correlations observed in October (r=-0.35; P˃0.05) and November (r=-0.47; P = 0.008), and only in the winter of 2020 was the correlation between THI and milk yield significantly positive again. The identified negative relationship between THI levels and milk yield in autumn under thermoneutral conditions warrants appropriate interpretation, while considering other indicators that may affect cow milk productivity.

As expected, a moderate negative relationship (r=-0.59, P = 0.043) was found between milk yield and the mastitis prevalence in the herd in this study. Additionally, a moderate positive correlation (r = 0.65, P = 0.022) was observed between milk yield and conception rate.

### Milk productivity of cows

**Fig. 2:**
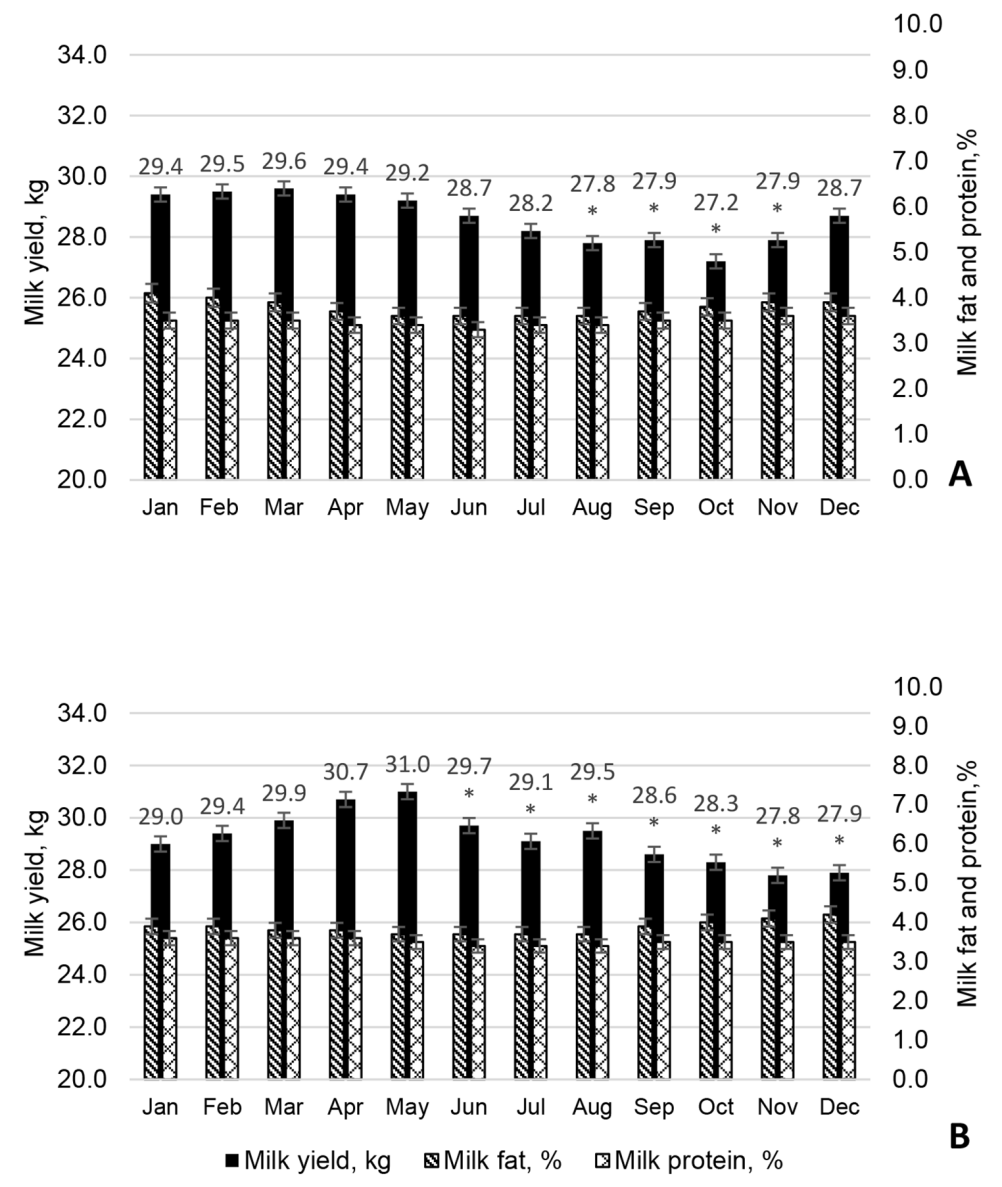
Milk production of Brown Swiss cows in 2019 (**A**) and 2020 (**B**). Significant differences (* P < 0.05) of milk yield for the herd of cows in the summer and autumn months are shown compared to May

During the summer and autumn months, the milk yield of cows (Fig. 1) was lower compared to May, which was considered the most comfortable period for dairy cows when kept in naturally ventilated barns all year round. Interestingly, the milk yield of BS cows (Fig. 2) during the summer heat decreased less (0.5–1.9 kg or 1.7–6.1%) than in autumn (2.0–3.2 kg or 6.8–10.3%).

### The prevalence of mastitis in the herd of BS cows

**Fig. 3:**
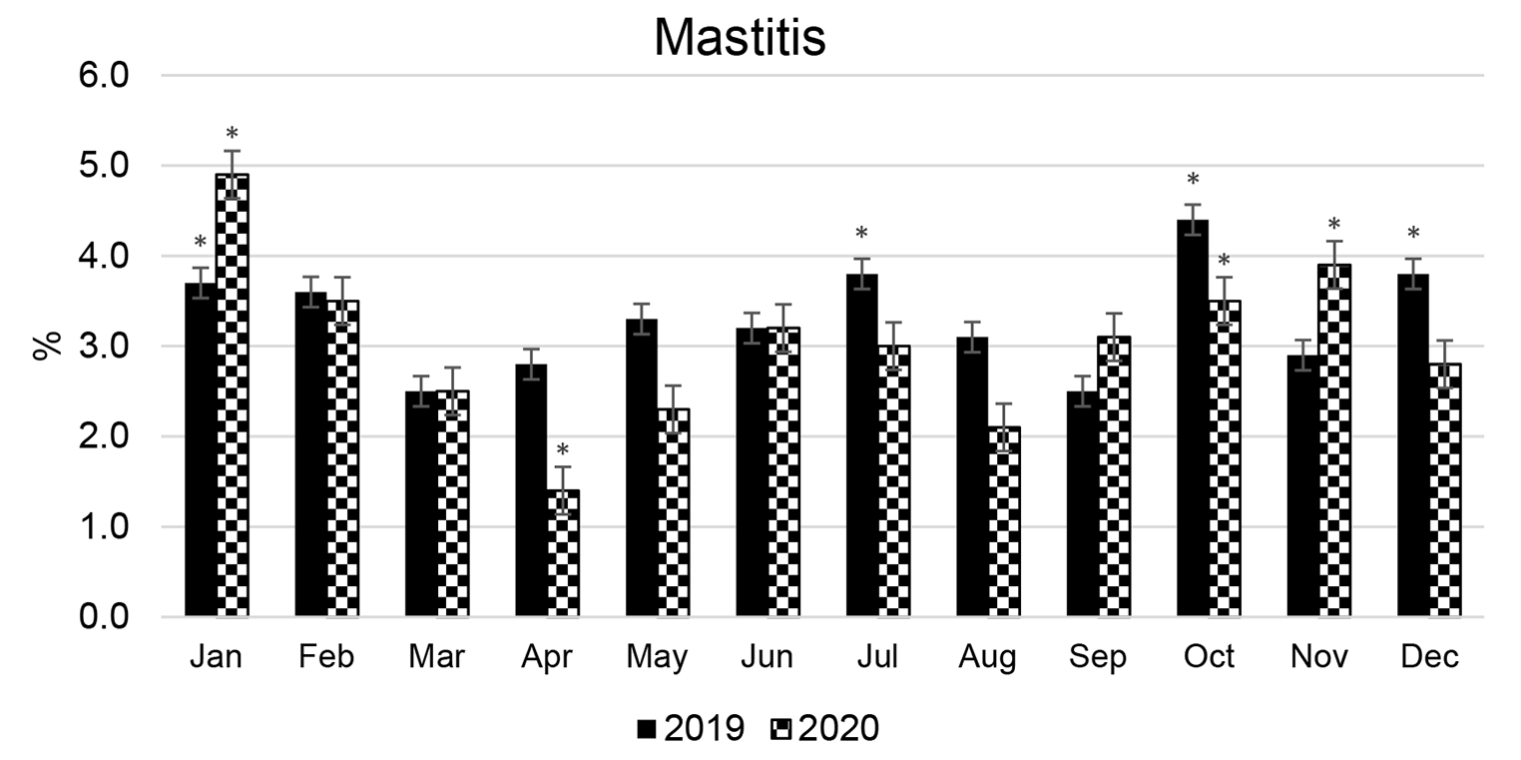
Manifestation of mastitis in a herd of Brown Swiss cows throughout the year. Significant differences (* P < 0.05) are compared with the average annual prevalenceof mastitis in the herd

Compared to the average annual mastitis prevalence in the same herd of cows in 2019 and 2020 (3.3% and 3.2%, respectively), it was established (Fig. 3) that cases of mastitis increased by 18–25% in October-November. Despite the increase in cases in July 2019 (by 15.2%), the incidence of mastitis in summer was even slightly lower than the average annual incidence, although the average THI values in summer were significantly higher than in autumn.

### Conception rate and number of calvings by herd of cows

**Fig. 4:**
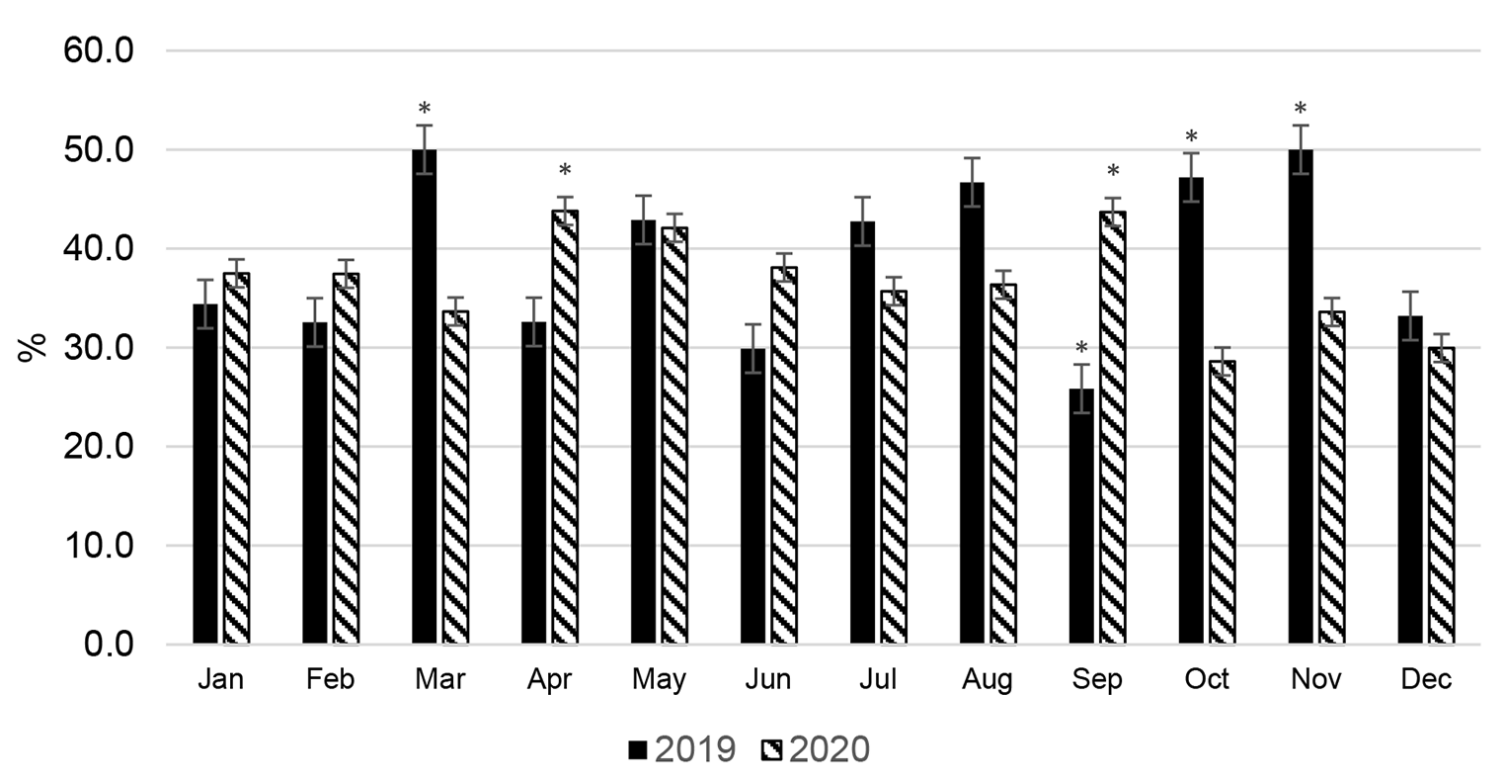
Conception rate of Brown Swiss cows during the year. Significant differences (* P < 0.05) are compared with the average annual percentage of cows’ conception rate in a herd

**Fig. 5:**
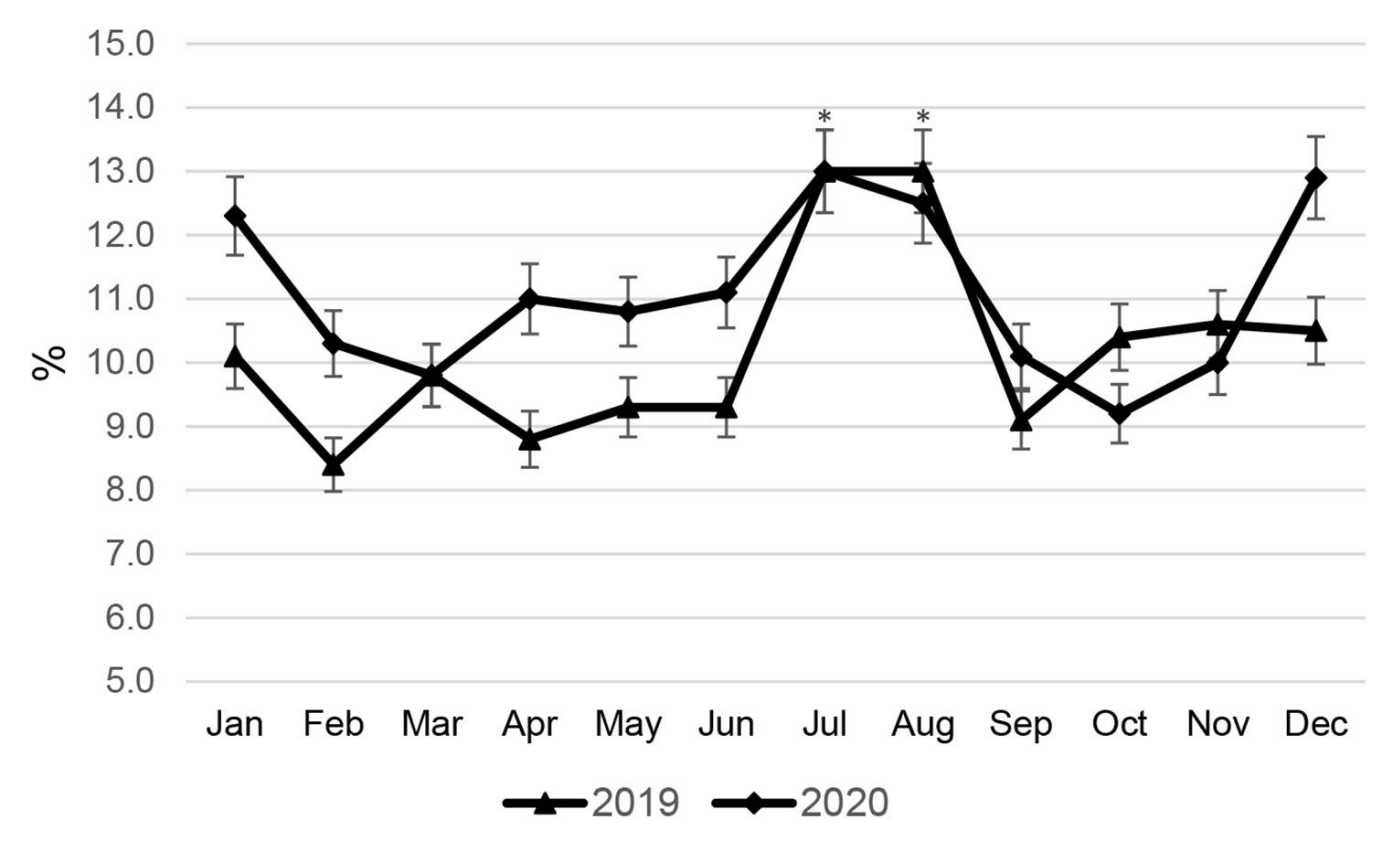
Percentage distribution of calvings of Brown Swiss cows throughout the year. Significant differences (* P < 0.05) are shown with respect to the average annual percentage of cows’ calving in a herd

The conception rate of cows (Fig. 4) and the number of calvings (Fig. 5) were monitored to assess the impact of seasonality on the reproductive ability and potential milk production of cows. The average conception rate for a herd of cows in 2019 and 2020 was found to be 39.0% and 38.1%, respectively. Both conception rate and number of calvings in the herd were distributed evenly throughout the year, with an average of 10.2% and 10.6% of cows calving each month in 2019 and 2020, respectively, based on the total number of cows on the dairy farm. It is rather challenging to determine the relationship between seasonality and animal reproduction and its potential impact on seasonal milk production from the data presented (see Figs. 4 and 5). The data presented in Figs. 2 and 3 reveal a problem associated with a drop in milk yield in BS cows in autumn and indicate a second peak of mastitis in the herd of cows in October, after the previous one in July, which may be a side effect of ALMYS. Additionally, there is a wave-like increase in the conception rate of cows in October-November 2019 and in September 2020 (Fig. 4), following a decrease in this indicator at the end of summer, which will cause more dry periods and calvings to occur during the hot period of the following year. Therefore, plenty of cows experienced the dry period and calving during summer HS (Fig. 5); according to our results, this could have a negative impact on the autumn milk yield of animals.

However, the factorial ANOVA, the purpose of which was to determine the percent of exposure (%) of the mentioned factors (conception rate, number of calvings and mastitis prevalence in the herd, as well as THI value) on milk yield of dairy cows did not confirm their significant influence on yield of BS cows. The total percentage of influence of these factors on cow milk yield was only 2.9% (Table 1). At the same time, only the joint influence (interaction) of such factors as “Conception rate” and “THI” on milk yield was significant, but extremely low (only 1.12%, P < 0.05).

**Table 1:** Percentage of exposure of factors “Mastitis prevalence (factor MAST)”, “Conception rate (CONC)”, “Number of calvings (CALV)”, and “Temperature-humidity index (THI)” on cow milk yield as determined by four-factor analysis of variance (ANOVA)

Factors	ANOVA parameters				
	SS	MS	F	p-value	$\eta _x^2,\%$
MAST	0.63	0.63	1.09	0.3244	0.12
CONC	0.00	0.00	0.00	0.9534	0.00
CALV	0.91	0.91	1.58	0.2409	0.17
THI	1.07	0.53	0.92	0.4318	0.20
Interaction, MASTxCONC	0.36	0.36	0.63	0.4478	0.07
Interaction, MASTx CALV	0.01	0.01	0.02	0.8934	0.00
Interaction, MASTxTHI	1.57	1.57	2.71	0.1339	0.29
Interaction, CONCx CALV	3.89	1.94	3.37	0.0810	0.72
Interaction, CONCxTHI	6.02	3.01	5.21	0.0314	1.12
Interaction, CALVxTHI	0.11	0.11	0.20	0.6677	0.02
Interaction, MASTxCONCx CALV	0.93	0.93	1.62	0.2353	0.17
Error	523.36	0.58			97.1

SS: the sum of squares; MS: mean square; F: Fisher criterion; p-value: the degree of probability of the result; $\eta _x^2,\%$: the percentage of influence of the studied factor

At the same time, the influence of the “Season” and “Year” factors on the milk yield of BS cows was significant (Table 2). The percentage of influence of these factors was 82.8% (P < 0.05). At the same time, seasonality had the greatest impact on cow milk yield (59%, P < 0.05).

**Table 2:** Percentage of exposure of factors “Season” and “Year” on cow milk yield determined by two-factor analysis of variance (ANOVA)

Factors	ANOVA parameters				
	SS	MS	F	p-value	$\eta _x^2,\%$
Season	12.28	4.09	18.40	0.0000	59.4
Year	2.28	2.28	10.25	0.0056	11.0
Interaction, Season x Year	2.57	0.86	3.24	0.0301	12.4
Error	3.56	0.22			17.2

SS: the sum of squares; MS: mean square; F: Fisher criterion; p-value: the degree of probability of the result; $\eta _x^2,\%$: the percentage of influence of the studied factor

**Fig. 6:**
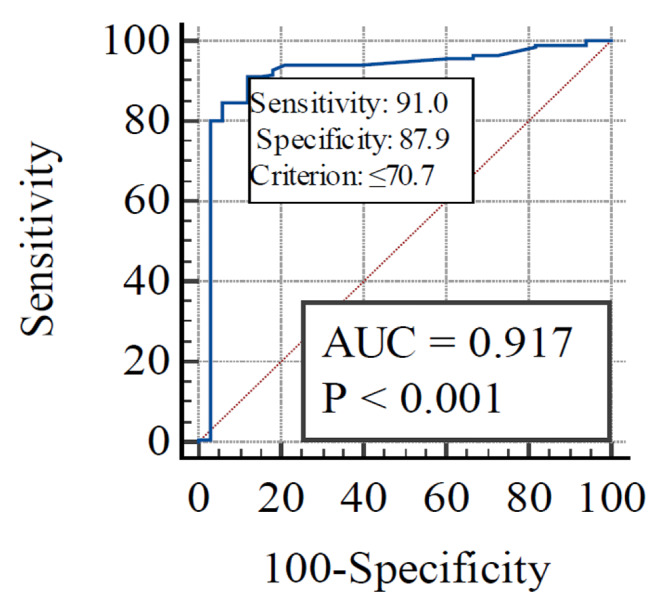
Parameters of the ROC-curve (AUC: area under ROC-curve) when determining the threshold of the temperature-humidity index, above which there was a drop-in milk yield in Brown Swiss cows

The threshold value of THI was also determined, the exceeding of which should be accompanied by a drop in daily milk yield (Fig. 6). Based on the results of the ROC-analysis, it was found that the threshold value for milk yield of BS cows was at the level of THI = 70.7 units. The sensitivity and specificity of the test were 91% and 88%, respectively (P < 0.01).

This means that with average daily THI values ≤ 70.7 units, 91% of cows will not experience a drop-in milk yield. If the threshold value is exceeded (THI > 70.7 units), 88% of cows will experience a decrease in milk yield. The quality of the model used was excellent (with AUC = 0.917).

## Discussion

The use of THI is a suitable approach for investigating the impact of weather conditions on dairy cows (Fodor et al. 2018). THI has been shown to have a significant association with milk production (Herbut et al. 2019) and physiological parameters during HS (Mader et al. 2006; Hoffmann et al. 2021), thereby enabling predictions of potential losses due to seasonal HS in dairy farms (Wangui et al. 2018).

There are diverse reasons for a herd-wide drop in milk yield. It is physiological that the milk yield of healthy cattle increases during the first three to six weeks of lactation, followed by a gradual and then sharp decline in performance, until milk secretion dries up or is discontinued by drying off (Macciotta et al. 2005). Due to a seasonal calving pattern and a simultaneous drying off at the end of the lactation, milk yield of the whole herd would decline in the same period.

One possible reason for the autumn decrease in milk yield in the BS cows may be the accumulated heat load in the previous summer, as also described by Amadori and Spelta (2021). Accordingly, the negative effects of HS can persist over long periods of time, even after returning to more favorable climatic conditions. Although HS appears to exhibit short-term mechanisms of action, it tends to maintain its effects over a long period of time, well beyond the actual period of the stress condition. This suggests that the reduction in milk yield of BS cows during the autumn should be considered as an outright manifestation of ALMYS, accompanied by an increased prevalence of mastitis among the cows of the herd during this period, despite thermoneutral conditions.

It is possible that the seasonal rhythm of wild animals still persists in domesticated cattle. The aurochs (*Bos primigenius*), for example, had a brief mating season in late summer, with calves born in late spring, resulting in a natural decrease in milk production during late summer/fall (Rokosz 1995). This aligns with a US study conducted by Salfer et al. (2019), who observed that milk production follows an annual rhythm and suggested that endogenous annual rhythms may account for up to a 3.3 kg/d difference in milk production across the year. Additionally, it is conceivable that dairy cows maintain their milk yield relatively constant even during HS, as milk production is intended to nourish the calf, which requires more milk on hot days. It is only after the heat period passes that the dairy cow reduces milk yield. Salfer et al. (2019) made similar assumptions and posited that lactating mammals likely produce more milk and more fat and protein for newborns during winter, when energetic demands are higher.

Moreover, the shortening of days during summer and fall, and the resulting decrease in daylight, also impacts milk production. This was supported by an experiment conducted in New Zealand dairy systems, which suggested that changes in photoperiod, mediated by increased concentrations of plasma melatonin and decreased concentrations of plasma prolactin, may contribute to some of the variation in the volume and quality of milk throughout the season (Auldist et al. 2006).

Recent research (Maggiolino et al. 2020; Mylostyvyi et al. 2021) suggests that BS cows are more heat-tolerant than Holstein cows. In addition, a recent study by Cuellar et al. (2023) suggests that BS breed regulated body temperature under heat stress better than HF, but BS breed were not more resistant to HS in terms of milk yield. There are probably genetic differences in thermotolerance that are independent of body temperature regulation.

In our study, we aimed to provide a plausible explanation for the autumn decline in milk yield in BS cows within the context of the adverse long-term effects of summer HS on milk yield, fertility, and mastitis in large commercial dairy operations. However, our findings posed a challenge to reconcile with prior research. Firstly, we observed differences in the time-course of milk yield between BS and HF cows. While HF cows show minimum production in summer, their milk production increases in October, albeit not as much as in spring (Amadori and Spelta 2021). In contrast, BS cows showed a further and significant decrease in milk yield in October, despite a sharp decline in THI values after September in both 2019 and 2020. Thus, under our experimental conditions, there was no direct impact of THI values on milk yield in late summer-autumn. Secondly, BS cows did not exhibit the near absence of fertilization during the summer period, which is usually observed in HF cows, at least in Southern Europe. This characteristic of BS cows results in a more balanced distribution of calvings throughout the year and likely leads to a lower prevalence of dry cows in summer compared to HF cows. That is, a lower percentage of cows undergo involution of the mammary gland under the negative influence of summer heat.

Overall, the critical issue concerning ALMYS is the identification of the long-term effector mechanisms linking HS and milk yield over several weeks. HS is a non-infectious stressor that can be sensed and countered by the innate immune system (Amadori 2016). The conceptual framework of “Trained Immunity,“ previously proposed by Amadori and Spelta (2021) to explain ALMYS, is consistent with the available data. “Trained Immunity” is a unique form of innate immune memory based on epigenetic changes in innate immunity genes that regulate chromatin accessibility, as previously demonstrated in dairy cows following bovine mastitis (Chang et al. 2015). Such epigenetic changes alter the response to the same or similar stressors upon subsequent exposure. In the case of ALMYS, “Trained Immunity” following HS would induce a significant shift in the metabolism of inflammatory cells, leading to higher basal glucose consumption, competing directly with milk synthesis.

There is substantial evidence of severe economic losses in dairy farms due to ALMYS. For example, in a dairy cattle farm of 100 lactating cows for 90 days, around 22.5 tons of milk would be lost annually (Amadori and Spelta 2021).

The negative correlation between milk production and THI (from − 0.538 to -0.899) during the warm season in a tropical climate is highly significant and understandable (Narmilan et al. 2021). However, the complex correlation between milk yield and THI values in the fall indirectly suggests that ALMYS is often overlooked in cows. A closer examination of previous linear regression models (Konyves et al. 2017) reveals that the correlation coefficient between THI and daily milk yield was higher in autumn (r=-0.529; R2 = 0.280) than in summer (r= -0.453; R2 = 0.205). This may indicate that cows, exhausted by summer HS, are more susceptible to environmental factors, even at low THI values in autumn. In addition, the long-term effects of summer HS related to changes in mammary gland cell function should be considered, which may be responsible for the decreased milk production in autumn (Tao et al. 2018).

There are breed differences between Holstein and BS cows in their response to thermal stress, as we reported in our recent study (Mylostyvyi et al. 2021). However, our study was limited to differences in the physiological response of cows of these breeds and their milk yield to summer HS. Similar studies were carried out in Italy (Maggiolino et al. 2020), and their results suggest that BS cows had higher thresholds for temperature-humidity index (THI) for milk yield drop than Holsteins during summer heat due to the lower ability of Holstein cows to correct their negative energy balance (Strączek et al. 2021). However, our study does not support the claim that BS cows tend to produce the same amount of milk with increasing THI but with worse components (Maggiolino et al. 2020). This lack of a THI threshold for BS cows in the study of Maggiolino et al. (2020) could be due to regional differences in climate, housing and cooling systems, and nutritional management.

The present study indicated that the THI threshold (70.7) for BS cows, above which daily milk yield decreases, is higher than the THI threshold for high-producing HF cows (THI = 68). Zimbelman et al. (2009) found that physiological parameters and milk yield were negatively affected at THI conditions above this threshold. This may indicate to some extent possible similar mechanisms for the manifestation of the ALMYS in these breeds. However, this result confirms that BS cows are more resistant to heat stress than HF cows (Mylostyvyi et al. 2021; Cuellar et al. 2023).

The lactation phase is likely to affect the level of HS tolerance. However, the available reports are contradictory and inconclusive. According to Brouček et al. (2009), cows are less able to cope with temperature stress during the first stage of lactation and immediately after calving. At the same time, cows in late lactation are also quite susceptible to HS (Heinicke et al. 2019). There are also reports that suggest that the monthly milk yield is highest during the spring and lowest in winter, with early and mid-lactating cows performing best in the spring, while late lactating cows perform better in the summer. Mid-lactating cows are most adversely affected by summer conditions, while early and late lactating cows are most affected by winter conditions (Perera et al. 1986). These data demonstrate that cows in different stages of lactation respond differently to environmental changes.

The strength of the relationship between the environment and milk yield depends on the level of milk productivity. Specifically, cows producing 25 kg or more of milk per day have a significantly stronger negative correlation between the sum of effective temperatures and milk yield (r = -0.304) than cows producing 20.1–24.9 kg of milk per day (r = -0.178) (Navratil and Falta 2017).

It is probable that the quantity of milk loss during the autumn season is related to the duration of heat waves. In their recent study, Maggiolino et al. (2022) observed that the extent of milk loss, including milk proteins, was dependent on the duration of heat waves surpassing the THI threshold values for BS cows. Additionally, primiparous appeared to have a less effective metabolic response to HS when compared to multiparous cows, likely due to incomplete growth processes overlapping milk production, making heat dissipation more challenging.

However, most of the studies conducted on Italian BS cows (Maggiolino et al. 2020, 2022) do not provide a reasonable explanation for seasonal changes in milk production. These studies were conducted in different herds, under varying conditions, and only during the summer seasons over a ten-year period. Long-term effects resulting from chronic HS were not taken into account either. In contrast, our data over a two-year period indicate a significant decrease in autumn milk yields in BS cows, which aligns with the study conducted by Amadori and Spelta (2021) on Italian Holstein cows. This was the main factor contributing to our hypothesis on the manifestation of ALMYS in Ukrainian BS cows. While there are numerous reports on the long-term effects of summer HS, they are not primarily presented in the context of the manifestation of ALMYS.

Summer HS can lead to negative energy balance and suppression of the immune system (Lacetera et al. 2005; Joo et al. 2021), which may contribute to diseases in dairy cows, such as mastitis (Tao et al. 2018; Vitali et al. 2020). However, individual and breed differences in response to HS should be taken into account. Joo et al. (2021) found differences in the immune response to HS in Jersey and Holstein cows, particularly with respect to peripheral blood mononuclear cells (PBMC) such as B cells and monocytes. While the biological mechanisms underlying such breed differences are not yet fully understood, Lacetera et al. (2006) have previously reported similarities in the immune response of PBMCs to HS in BS and HF cows. Strong evidence suggests fundamental differences in the innate immune response between HF and BS cows. Under the same in vitro conditions, the macrophages of BS cows produce significantly more reactive nitrogen species (RNS) and less Interleukin (IL)-1ß (inflammasome response), compared to cells from HF cattle (Gibson et al. 2016). These differences could lead to different profiles of “Trained Immunity” and potentially account for the peculiarities of ALMYS in the two cattle breeds. It should be noted, however, that most studies on Italian BS cows do not account for seasonal changes in milk production or the long-term effects associated with chronic HS, and thus further research is needed in this area.

Overall, two general conclusions can be drawn from the research:

1. ALMYS does occur in BS cows, albeit with some peculiarities compared to HF cattle.
2. ALMYS cannot be solely attributed to heat stress’s direct, short-term impact. Therefore, it is likely that this stressor triggers long-term mechanisms even after THI values have returned to thermoneutral conditions.

## Data Availability

The datasets generated during and/or analysed during the current study are available from the corresponding author on reasonable request.
